# Etanercept-associated Nephropathy

**DOI:** 10.7759/cureus.5419

**Published:** 2019-08-18

**Authors:** Awais Ammar, Hafiz Zafar Ahmed Mahmood, Zainab Shahid, Rohit Jain, Guoli Chen

**Affiliations:** 1 Internal Medicine, Penn State Health Milton S. Hershey Medical Center, Hershey, USA; 2 Internal Medicine, Lake Erie College of Osteopathic Medicine, Erie, USA; 3 Pathology, Penn State Health Milton S. Hershey Medical Center, Hershey, USA

**Keywords:** anti-tnf therapy, anti tnf, rheumatoid arthritis, etanercept, tnf inhibitor, tnf-tumor necrosis factor, tumor necrosis factor alpha (tnf alpha), glomerulo nephritis, acute kidney injury

## Abstract

Anti-TNF (tumor necrosis factor) medications work by inhibiting the production of TNF or its effect on target organs. TNF is a cell-signaling protein, or cytokine, involved in systemic inflammation and is one of the cytokines that make up the acute phase reactants. TNF inhibitors are available for the treatment of a number of rheumatic and other immune-mediated diseases. Treatment of rheumatoid arthritis with anti-TNFα (tumor necrosis factor-alpha) agents may lead to autoantibody formation and flares of vasculitis. Although medications are a common cause of renal injury, anti-TNFα medications very rarely cause renal complications. We present a case of a patient who presented with nausea and flu-like illness and was ultimately found to have etanercept-induced nephropathy.

## Introduction

Anti-TNF (tumor necrosis factor) medications work by inhibiting the production of TNF or its effect on target organs. TNF is a cell-signaling protein, or cytokine, involved in systemic inflammation and is one of the cytokines that make up the acute phase reactants. TNF is produced chiefly by activated macrophages, although it can be produced by other cells such as CD4+ (cluster of differentiation 4) lymphocytes, natural killer (NK) cells, neutrophils, mast cells, eosinophils, and neurons [1]. TNF levels are increased in a variety of immune-mediated diseases, such as rheumatoid arthritis, polyarticular juvenile idiopathic arthritis, and ankylosing spondylitis, and therefore TNF inhibitors are used in the treatment of these diseases.

Etanercept, otherwise known as enbrel, is a TNF inhibitor that was approved by the Food and Drug Administration (FDA) in 1998 for the treatment of rheumatoid arthritis, polyarticular juvenile idiopathic arthritis in patients aged two or older, psoriatic arthritis, ankylosing spondylitis, and plaque psoriasis in patients aged four or older [2]. The most common adverse reactions reported with this medication are infections and injection site reactions. Nephrotoxicity is a rare side effect of etanercept and few reports of this occurrence have been described in the literature [3-7].

## Case presentation

A 56-year-old female with a past medical history significant for hypothyroidism, hyperlipidemia, and seropositive rheumatoid arthritis diagnosed in 2014 presented to the nephrology clinic upon referral for evaluation of acute kidney injury, proteinuria, and hematuria. The patient first presented to her primary care physician in July 2017 due to a flu-like illness accompanied by nausea, stomach upset, and dark urine. She was prescribed a two-week course of ciprofloxacin by her primary care physician for a presumed urinary tract infection and then prescribed a two-week course of trimethoprim-sulfamethoxazole due to persistent symptoms.

In August 2017, the patient presented to the emergency department with persistent nausea, vomiting, fatigue, and anorexia. Laboratory workup at this visit revealed an elevated creatinine of 1.79 mg/dL from a baseline creatinine of 0.75 mg/dL in February 2017. The patient received intravenous (IV) fluids and ondansetron. Further workup was non-contributory, and the patient was subsequently discharged to home with a follow-up appointment with her primary care physician. The following month, the patient presented to the emergency department with similar symptoms and was admitted to the hospital. Her creatinine upon presentation was 2.00 mg/dL, which improved to 1.56 mg/dL with IV fluids. She was discharged to home the following day with a plan to follow up with outpatient nephrology.

The patient presented to the nephrology clinic in September 2017 with persistent generalized fatigue, malaise, nausea, and occasional vomiting. She reported that her lack of energy affected her ability to perform her job. The patient’s medications included levothyroxine 75 mcg daily, methotrexate 7.5 mg per week, etanercept 50 mg subcutaneous every week since January 2016, simvastatin 20 mg daily, famotidine, folic acid, vitamin D and biotin. Vital signs revealed a temperature of 37°C, a pulse of 72 beats per minute, a blood pressure of 130/92, and a respiratory rate of 18. Physical examination did not reveal any abnormalities. Initial laboratory workup was significant for a blood urea nitrogen (BUN) level of 29 mg/dL (normal 7-20 mg/dL), creatinine level of 1.67 mg/dL (normal 0.6-1.0 mg/dL) and a urine protein/creatinine ratio of 3.30 (normal <0.5) (Table 1). Additional workup revealed a positive antinuclear antibody, anti-SSA (Sjögren’s syndrome antigen A) antibody, anti-SSB (Sjögren’s syndrome antigen B) antibody, and anti-histone antibody (Table 2).

**Table 1:** Initial Laboratory Workup BUN: Blood urea nitrogen; Prot/Cret: Protein/Creatinine.

Lab	Result
BUN	29 mg/dL (normal 7-20 mg/dL)
Creatinine	1.67 mg/dL (normal 0.6-1.0 mg/dL)
Hemoglobin	12.4 g/dL (normal 11.7-15 g/dL)
Platelets	276 K/uL (normal 150-350 K/uL)
Albumin	3.4 g/dL (normal 3.5-5.2 g/dL)
Urine Prot/Cret Ratio	3.30 (normal <0.5)

**Table 2:** Additional Workup Anti-SSA: Anti-Sjögren’s syndrome antigen A; Anti-SSB: Anti-Sjögren's syndrome antigen B; Anti-dsDNA: Anti-double stranded deoxyribonucleic acid; Anti-RNP: Anti-ribonucleoprotein; Anti-Jo-1: Anti-histidyl tRNA (transfer ribonucleic acid) synthetase; ANCA: Anti-neutrophil cytoplasmic antibody.

Lab	Result
Anti-nuclear antibody	+VE 1:640 SPECKLED PATTERN (normal <1:80 titer)
Anti-SSA antibody	97.85 U/mL (normal <20 U/mL)
Anti-SSB antibody	56.89 U/mL (normal <20 U/mL)
Anti-histone antibody	1.54 U/mL (normal <1 U/mL)
Anti-dsDNA antibody	Normal (normal <30 IU/mL)
Anti-Smith antibody	Normal (normal <20 U/mL)
Anti-RNP antibody	Normal (normal <20 U/mL)
Anti-centromere antibody	Normal (normal <20 U/mL)
Anti-proteinase antibody	Normal (normal <19 AU/mL)
Anti-scleroderma 70 antibody	Normal (normal <20 U/mL)
Anti-Jo-1 antibody	Normal (normal <20 U/mL)
ANCA screen	Negative (normal <20 titer)
Serum kappa/lambda ratio	1.32 mg/dL (normal 0.26-2.65 mg/dL)
Serum protein electrophoresis	Normal
Urine immunofixation and electrophoresis	Normal

**Figure 1:**
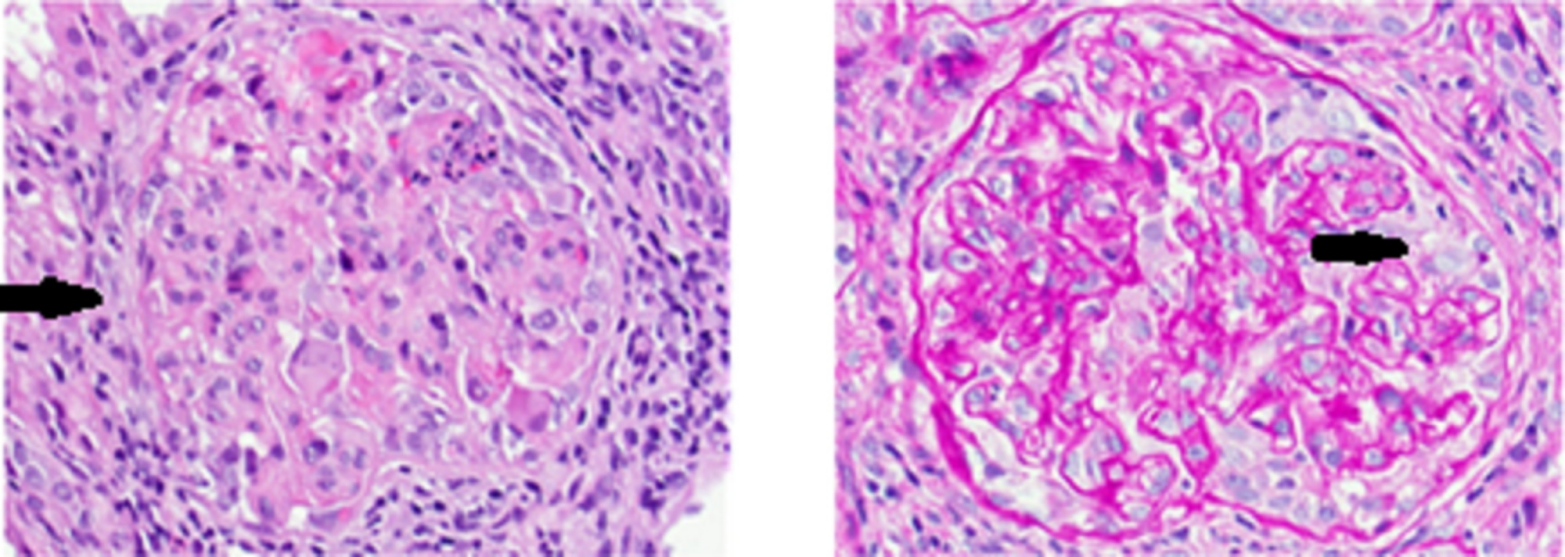
Light Microscopy Light microscopy depicting moderate interstitial fibrosis with associated tubular atrophy and chronic inflammation.

**Figure 2:**
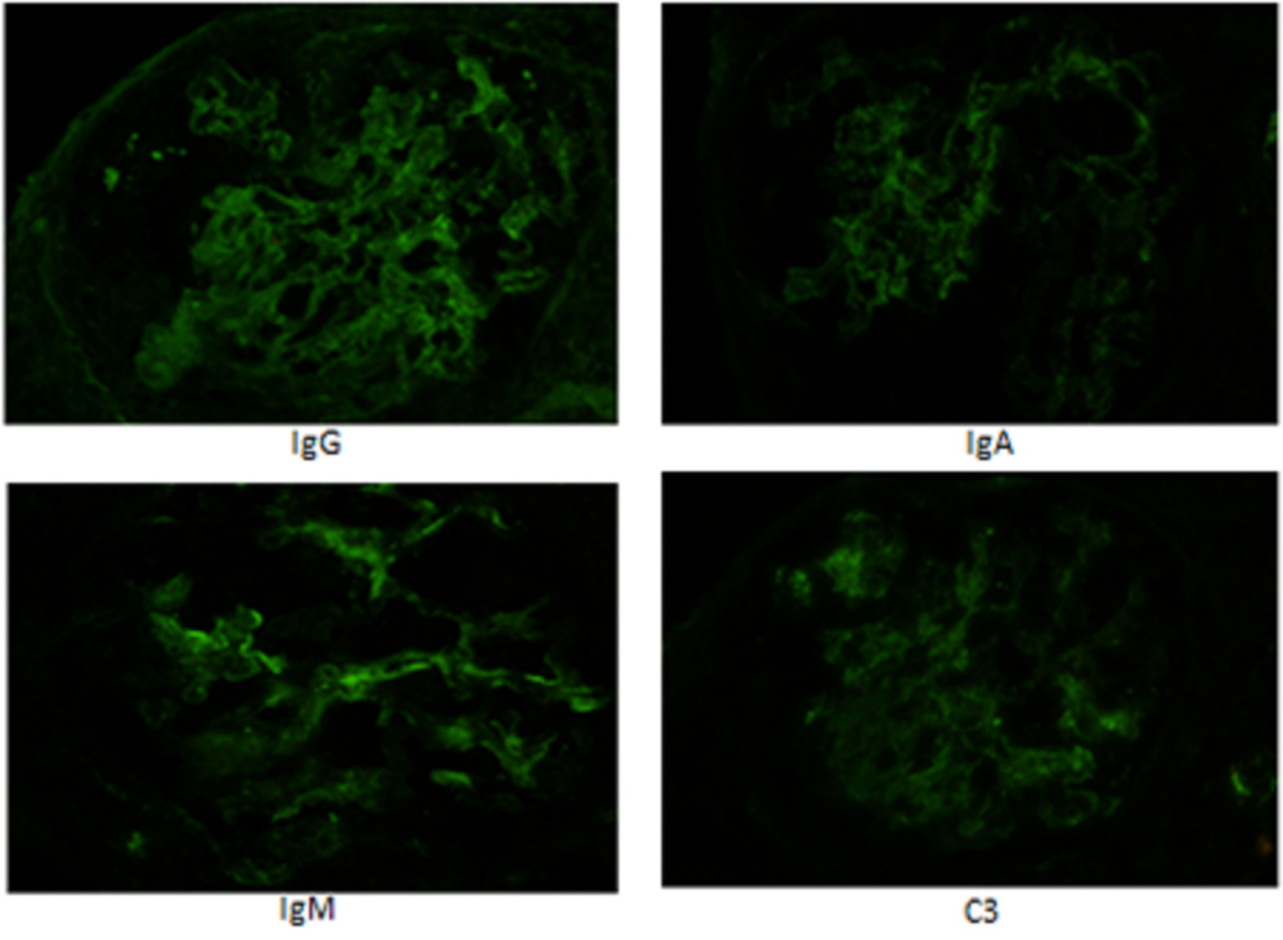
Immunofluorescence Immunofluorescence depicting granular staining of IgG (3+), IgA (2+), IgM (2+), and C3 (2+) in the glomerular walls and mesangium. IgG: Immunoglobulin G; IgM: Immunoglobulin M; IgA: Immunoglobulin A; C3: Complement component 3.

**Figure 3:**
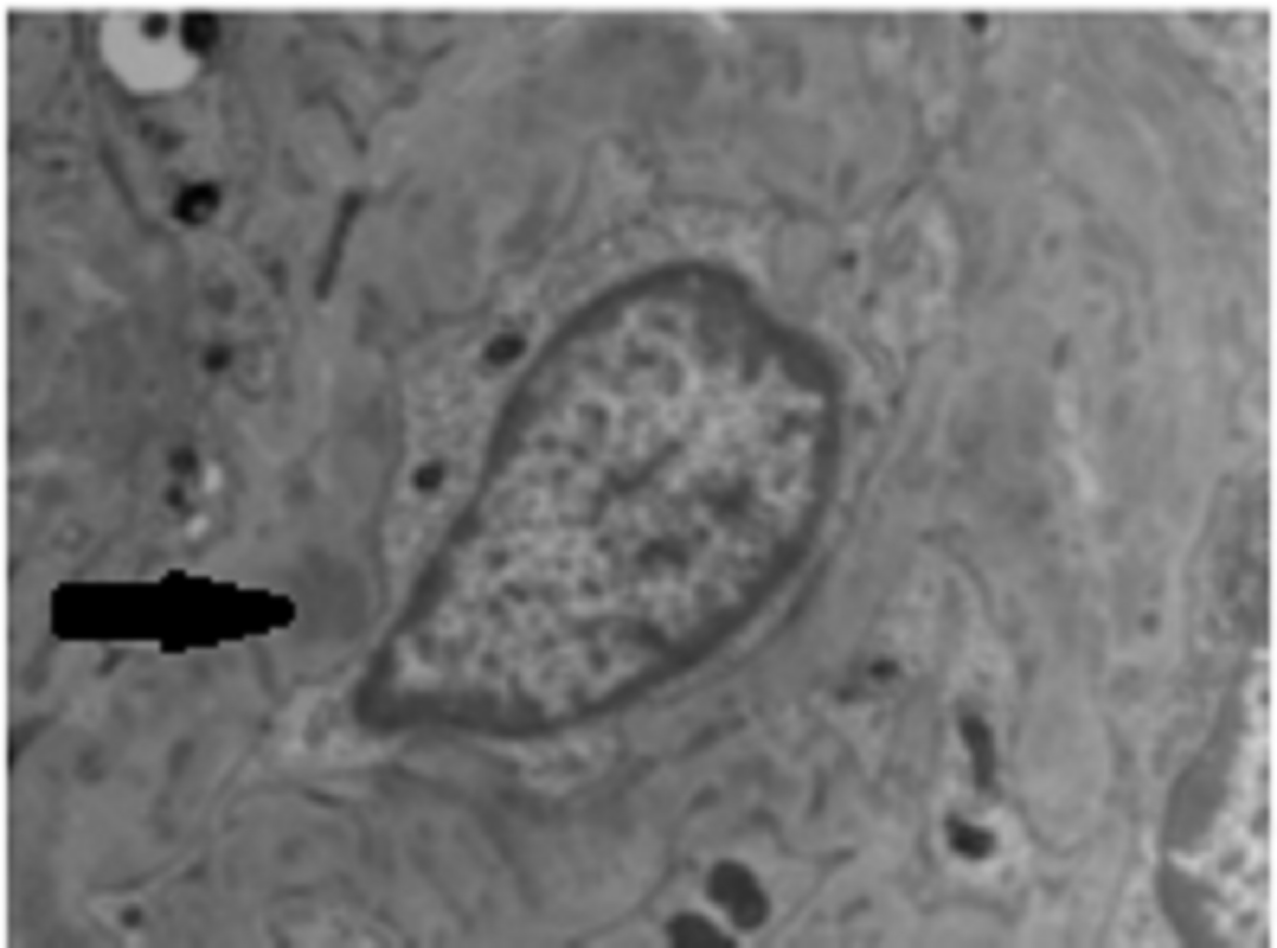
Electron Microscopy Electron microscopy depicting scattered electron densities predominantly in the intramembranous and mesangial areas (black arrow).

**Figure 4:**
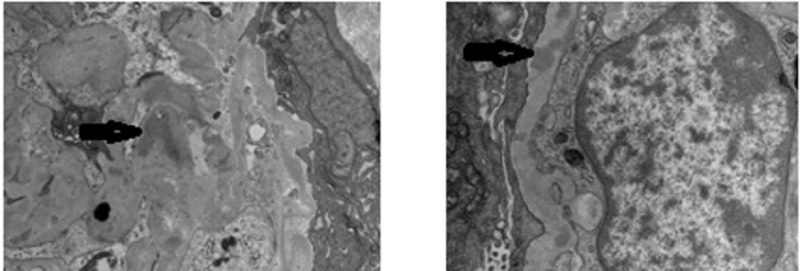
Electron Microscopy Electron microscopy depicting scattered electron densities predominantly in the intramembranous and mesangial areas (black arrows).

The patient agreed to a kidney biopsy for further evaluation, and the renal specimen was examined under light microscopy, electron microscopy, and immunofluorescence. Under light microscopy, four out of 14 glomeruli were globally sclerotic and at least six showed fibrocellular crescents, most of which were circumferential. There was moderate interstitial fibrosis and associated tubular atrophy as well as chronic inflammation involving approximately 30-40% of the cortex (Figure 1). Under immunofluorescence, some of the 14 glomeruli showed crescentic formation and the glomerular walls and mesangium, as well as some vessels, had granular staining of IgG (3+), IgA (2+), IgM (2+), C3 (2+), kappa (3+) and lambda (2 to 3+) light chains (Figure 2). Electron microscopy of the specimen revealed crescentic changes, highly distorted capillary tufts, extensive foot process effacement on the capillary surface, and scattered electron densities predominantly in the intramembranous and mesangial areas and rarely in the subendothelial and subepithelial areas (Figures 3, 4).

**Figure 5:**
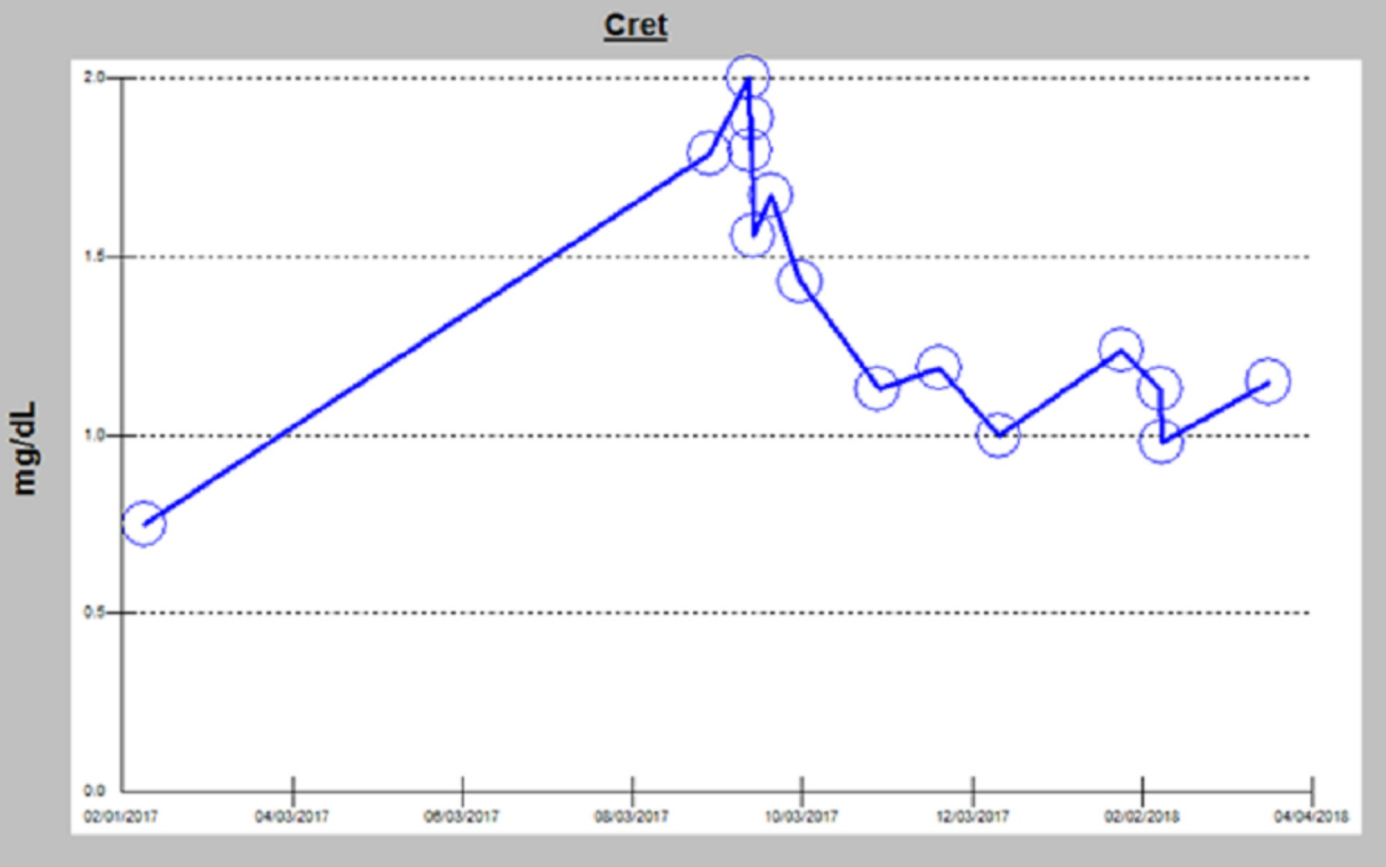
Creatinine Trend Creatinine trend after withdrawing etanercept and beginning treatment with mycophenolate mofetil and steroids.

The biopsy results were significant for crescentic necrotizing glomerulonephritis with moderate interstitial fibrosis and moderate atherosclerosis. The patient was diagnosed with crescentic glomerulonephritis, attributed to etanercept in the clinical context as a diagnosis of exclusion. The etanercept was subsequently discontinued and the patient was prescribed steroids and mycophenolate mofetil. The patient was closely followed up in the clinic and her creatinine gradually improved after stopping etanercept and beginning steroids and mycophenolate mofetil (Figure 5). Her most recent laboratory results showed a near-normal creatinine level.

## Discussion

Medications are a common cause of renal injury, and the three main cell types within the glomerulus which are targeted by medications to cause nephrotoxicity are the visceral epithelial cells, endothelial cells, and mesangial cells. These cells are either directly targeted by a medication or by its metabolites, or alternatively injured due to a modification in the immune system caused by certain medications. There are many drugs which have shown an association with renal injury. Interferon (IFN) alpha and beta, pamidronate, and lithium have been shown to cause both minimal change disease and focal segmental glomerulosclerosis. Additionally, non-steroidal anti-inflammatories and cyclooxygenase-2 inhibitors have been shown to cause minimal change disease, and sirolimus and anabolic steroids have been shown to cause focal segmental glomerulosclerosis. Certain anti-angiogenesis drugs, such as mitomycin-C, gemcitabine, interferon, antiplatelet agents, and quinine have been shown to cause thrombotic microangiopathy [8].

There are currently five TNF inhibitors available for the treatment of rheumatoid arthritis, psoriatic arthritis and psoriasis, spondylarthritis, inflammatory bowel disease, and a number of other rheumatic and other immune-mediated diseases. Biosimilar forms of several TNF inhibitors have become commercially available in some countries, and additional biosimilar agents are being developed. These medications include etanercept, infliximab, adalimumab, certolizumab, and golimumab. Etanercept is a soluble p75 TNF receptor fusion protein that consists of two p75 TNF receptors bound to the Fc portion of immunoglobulin G (IgG). TNF is considered a key inflammatory cytokine in the pathogenesis of rheumatoid arthritis and its dysregulated secretion both induces and maintains synovitis in patients with this disease. As such, TNF inhibitors such as etanercept are widely used in the treatment of this disease [1].

Studies suggest that TNF inhibitors work by causing cell cycle arrest, apoptosis, and suppression of cytokine production in both monocytes and macrophages in synovial fluid and blood [9]. In addition, they also reduce synovial expression of chemokines and reduce inflammatory cell migration to the synovium [10]. There have been very few reports of nephrotoxicity caused by TNF inhibitors. These include a case of extra capillary glomerulonephritis in a pediatric patient being treated with etanercept for juvenile psoriatic arthritis, two cases of acute kidney injury and sarcoid-like granulomatosis in patients being treated with etanercept for rheumatoid arthritis, a case of minimal change disease in a patient being treated with etanercept for psoriasis, and a case of membranous glomerulonephritis in a patient being treated with etanercept for ankylosing spondylitis [3-7]. One study on five patients with longstanding rheumatoid arthritis suggested a pathogenic role of TNF inhibitor therapy for the development of glomerular disease due to a close temporal relationship with the onset of disease and both clinical and laboratory improvement after the withdrawal of TNF therapy [11].

Baseline testing for antinuclear antibody (ANA), anti-dsDNA (double-stranded DNA), and antineutrophil cytoplasmic antibody (ANCA) should be performed in all patients with rheumatoid arthritis prior to commencing etanercept inhibitor therapy in order to help identify new, drug-induced autoimmune processes in those patients who eventually develop glomerular disease. TNF inhibitors such as etanercept should be used with caution and patients on this therapy should have their renal function routinely monitored. Although it is rare, TNF inhibitor-induced nephrotoxicity should be considered in patients who are using TNF inhibitors and present with acute kidney injury and abnormal urinalysis. If patients do develop renal disease, a final decision about drug continuation or withdrawal should be entertained after discussing the risks of renal failure and the benefits of treatment of autoimmune disease with the patient.

## Conclusions

We present a rare case of etanercept-related crescentic glomerulonephritis in a patient being treated for rheumatoid arthritis. While the exact mechanism of this adverse reaction is still under investigation, TNF inhibitor-related nephrotoxicity is supported by the temporal relationship between onset of glomerular disease in patients without previous renal disease and improvement after the withdrawal of TNF inhibitor therapy.
